# ChemProspector and generic structures: advanced mining and searching of chemical content

**DOI:** 10.1186/1758-2946-4-S1-O17

**Published:** 2012-05-01

**Authors:** Valentina Eigner-Pitto, Josef Eiblmaier, Hans Kraut, Larisa Isenko, Heinz Saller, Peter Loew

**Affiliations:** 1 InfoChem GmbH, München, 81241, Germany

## 

Chemical information mining has turned into a well-established scientific area over the last five years. Several software solutions exist that are able to identify and extract names of chemical compounds in text documents and convert them into chemical structure-searchable information. Likewise, several programs exist which recognize chemical structures from images and translate them into the computer-readable format, the connection table. However, a still unsolved issue is the automatic abstraction of generic compounds (Markush structures). These usually consist of a core structure image and variable groups specified in the text, in additional images or in tables.

This presentation describes our hybrid approach to extract generic structure information from documents by using combining information science, cheminformatics, computational linguistics and pattern recognition techniques. Experiences with the envisaged methodology and the first results are presented.

This research project is funded by the German Ministry of Economics and Technology. It is part of the THESEUS research programme which has the goal to facilitate access to information, combine data to form new kinds of knowledge and lay the groundwork for new services on the Internet.

